# Previous Vaccination History and Psychological Factors as Significant Predictors of Willingness to Receive Mpox Vaccination and a Favorable Attitude towards Compulsory Vaccination

**DOI:** 10.3390/vaccines11050897

**Published:** 2023-04-25

**Authors:** Haneen Mahameed, Kholoud Al-Mahzoum, Lana A. AlRaie, Razan Aburumman, Hala Al-Naimat, Sakher Alhiary, Muna Barakat, Ala’a B. Al-Tammemi, Nesreen A. Salim, Malik Sallam

**Affiliations:** 1 Department of Pathology, Microbiology and Forensic Medicine, School of Medicine, The University of Jordan, Amman 11942, Jordan; 2 School of Medicine, The University of Jordan, Amman 11942, Jordan; 3 Jordan University Hospital, Amman 11942, Jordanrza0161688@ju.edu.jo (R.A.); 4 Nursing Department, Jordan University Hospital, Amman 11942, Jordan; s.hiary@ju.edu.jo; 5 Department of Clinical Pharmacy and Therapeutics, Faculty of Pharmacy, Applied Science Private University, Amman 11931, Jordan; 6 MEU Research Unit, Middle East University, Amman 11831, Jordan; 7 Migration Health Division, International Organization for Migration (IOM), The UN Migration Agency, Amman 11953, Jordan; 8 Prosthodontic Department, School of Dentistry, The University of Jordan, Amman 11942, Jordan; 9 Prosthodontic Department, Jordan University Hospital, Amman 11942, Jordan; 10 Department of Clinical Laboratories and Forensic Medicine, Jordan University Hospital, Amman 11942, Jordan; 11 Department of Translational Medicine, Faculty of Medicine, Lund University, 22184 Malmö, Sweden

**Keywords:** vaccine readiness, emerging infection, vaccine resistance, vaccine mandate, vaccination policy, voluntary vaccination

## Abstract

During the ongoing multi-country monkeypox (Mpox) outbreak, healthcare workers (HCWs) have represented a key group in mitigating disease spread. The current study aimed to evaluate the attitude of nurses and physicians in Jordan towards Mpox vaccination, as well as their attitude towards compulsory vaccination against coronavirus disease 2019 (COVID-19), influenza, and Mpox. An online survey was distributed in January 2023 based on the previously validated 5C scale for psychological determinants of vaccination. Previous vaccination behavior was assessed by inquiring about the history of getting the primary and booster COVID-19 vaccination, influenza vaccine uptake during COVID-19, and any history of influenza vaccine uptake. The study sample consisted of 495 respondents: nurses (*n* = 302, 61.0%) and physicians (*n* = 193, 39.0%). Four hundred and thirty respondents (86.9%) had heard of Mpox before the study, and formed the final sample considered for Mpox knowledge analysis. Deficiencies in Mpox knowledge were reflected in a mean knowledge score of 13.3 ± 2.7 (out of 20.0 as the maximum score), with significantly lower knowledge among nurses and females. The intention to receive Mpox vaccination was reported by 28.9% of the participants (*n* = 143), while 33.3% were hesitant (*n* = 165), and 37.8% were resistant (*n* = 187). In multivariate analysis, Mpox vaccine acceptance was significantly associated with previous vaccination behavior, reflected in higher vaccine uptake and with higher 5C scores, while Mpox knowledge was not correlated with Mpox vaccination intention. The overall attitude towards compulsory vaccination was neutral, while a favorable attitude towards compulsory vaccination was associated with higher 5C scores and a history of previous vaccination uptake. The current study showed a low intention to get Mpox vaccination in a sample of nurses and physicians practicing in Jordan. The psychological factors and previous vaccination behavior appeared as the most significant determinants of Mpox vaccine acceptance and of attitudes towards compulsory vaccination. The consideration of these factors is central to policies and strategies aiming to promote vaccination among health professionals in efforts to prepare for future infectious disease epidemics.

## 1. Introduction

In recent years, the study of the potential factors linked with the readiness to get vaccinated is gaining increasing interest [1]. This can be linked to the accelerated reporting of emerging infections with the need for vaccination as a central protective measure to control infectious disease spread and to reduce its associated morbidity and mortality [2,3]. Vaccination hesitancy, defined as the reluctance to be vaccinated despite the availability of vaccination services, represents a major global public health challenge [4,5]. It was listed among the top ten global health threats by the World Health Organization (WHO) in 2019, just before the emergence of the coronavirus disease 2019 (COVID-19) pandemic [6].

Vaccination hesitancy was studied extensively during the COVID-19 pandemic [1,7,8]. This research field gained momentum due to widespread public attention to the pandemic and the rapid availability of vaccines with concerns regarding their efficacy and safety, together with the circulation of misinformation regarding the virus and its vaccines [1,9,10]. Thus, COVID-19 vaccine hesitancy was a notable phenomenon with a high prevalence in many world regions [7,8,11].

The study of the factors linked to the willingness to get vaccinated can be helpful for future vaccine promotion strategies [12]. Analysis of vaccination hesitancy/rejection and its associated factors can help to devise well-informed intervention and educational measures aiming to promote vaccination, with subsequent positive effects reflected by proper control of infectious disease spread through population immunity [13,14,15]. The first critical step to address vaccination hesitancy is the accurate depiction of its scope [16]. This entails the accurate measurement of the factors associated with this widely prevalent phenomenon [17]. In turn, such an approach can help to develop cost-effective evidence-based tools tailored to the needs of the vaccine-hesitant populations (e.g., parents, health professionals) [15,18]. Therefore, the development and use of validated tools to measure vaccine hesitancy is of the utmost value [17,19].

One of the commonly used validated instruments to measure the determinants of vaccine hesitancy is the 5C scale developed by Betsch et al. [20,21]. To understand the driving factors behind the decision to receive a vaccine, the 5C model scrutinizes the following factors: (1) Confidence, which implies trust in vaccine safety and efficacy, and trust in health institutions and professionals [22]; (2) Collective responsibility, which involves feeling the need to protect the vulnerable groups in society from the potential harms of infectious diseases [23]; (3) Complacency, which involves the assessment of the perception of disease risks and its subsequent effect on perception of the necessity for vaccination [24]; (4) Constraints, which involves the assessment of the factors that could make vaccination an inconvenient experience (these factors include the ease of access to vaccination services in time and place, as well as the need to pay for vaccination) [4]; (5) Calculation, which entails the degree to which an individual is weighing the risks and benefits of vaccination besides the need to understand several aspects related to vaccination [20,21,25].

The emergence of monkeypox (Mpox) as a global outbreak in May 2022 was an example highlighting the importance of infectious disease surveillance [26,27,28]. Additionally, the Mpox outbreak underlined the need for continued progress to develop novel treatment and preventive approaches to tackle infectious disease threats [26,29]. Vaccines have been approved to prevent Mpox; however, this availability does not guarantee Mpox vaccine uptake among the most-at-risk groups which include men who have sex with other men (MSM), immunodeficient patients and healthcare workers (HCWs) [28,30,31,32,33].

Monkeypox is an emerging disease caused by the Mpox virus (MPXV), which has been endemic in Central and Western Africa, with occasional outbreaks outside the endemic areas [26,34,35]. The clinical manifestations of Mpox are similar to those of smallpox, albeit less severe and with lower mortality [29]. Antiviral drugs are available for the treatment of severe cases, and vaccines are also available and recommended for high-risk groups, as stated earlier, including HCWs at risk of repeated exposure [29,32].

In Middle East countries, very low prevalence of Mpox has been reported, with a single confirmed case in Jordan as of 22 March 2023 [36]. Nevertheless, vigilant surveillance and outbreak response measures (e.g., education and training of health professionals and vaccination of most-at-risk groups) are needed to contain potential virus introduction and subsequent community spread [30,37].

The role of HCWs’ knowledge and awareness cannot be overstated in the response to emerging infections’ threats [38]. This issue was of particular relevance during the 2022 Mpox outbreak, considering the previous and recent evidence suggesting the presence of noticeable knowledge gaps regarding Mpox among HCWs in various world regions [39,40,41,42,43,44]. Additionally, Mpox emerged in close proximity in time to the outbreak of COVID-19 and the associated circulation of misinformation and conspiratorial ideas regarding emerging infections, which were feared even before the emergence of COVID-19 [45,46,47]. Furthermore, the dynamic nature of vaccine hesitancy requires the continuous assessment of its scope and determinants [17]. This is particularly important among health professionals, considering their influential role in establishing community trust regarding vaccination [22,48,49]. Additionally, HCWs can have a primary role in community education and help to alleviate anxiety and fear that arise in response to emerging infections [50,51,52,53,54]. Therefore, the ongoing Mpox outbreak can provide an opportunity to study vaccination readiness, serving as a model enabling an understanding of the factors that might hinder vaccine acceptance for an emerging virus infection [1,55]. Furthermore, evidence continues to emerge indicating that the acceptance of Mpox vaccination is low among health professionals [56,57]. Thus, the study of the intention to get vaccinated among health professionals can help in the identification of underlying factors that may be targeted for proper control of the ongoing Mpox outbreak and for immediate response to future outbreaks.

A controversial strategy to promote vaccination is to introduce coercive measures that require compulsory (mandatory) vaccination [58]. This strategy has undeniable benefits in terms of achieving population immunity levels and in the prevention of outbreaks [59]. Nevertheless, ethical concerns accompany compulsory vaccination policies including the issues of informed consent and respecting individual autonomy; these should be balanced with the collective community benefit, particularly in the case of health professionals, who are responsible for providing care to vulnerable groups [59,60,61,62].

Therefore, the current study aimed to evaluate the willingness of nurses and physicians in Jordan to receive Mpox vaccination, while also exploring the possible associated factors influencing vaccine hesitancy/rejection. In addition, the attitudes towards compulsory vaccination against COVID-19, influenza, and Mpox were assessed.

## 2. Materials and Methods

### 2.1. Study Design, Settings and Ethics Statement

This study was based on a self-administered online questionnaire using a cross-sectional design.

A minimum required sample size was determined at 383 based on the following factors: a total of 55,000 nurses and physicians in Jordan with a ratio of 1.5:1 [63], with an estimated proportion of vaccine acceptance at 0.50, desired precision of ±0.05, and confidence level of 0.95 [64].

The survey instrument was based on previous literature addressing Mpox knowledge, the 5C psychological antecedents of vaccination (including an instrument validated in Arabic) and attitudes towards compulsory vaccination [20,21,41,65,66,67].

The questionnaire was created in Google Forms, and to expedite the process, the survey distribution was convenience-based, using a snowball sampling approach starting with the contacts of the authors and with encouragement of further distribution of the survey to their contacts. Survey distribution was undertaken on several social media and instant messaging platforms including Facebook, WhatsApp, Messenger, LinkedIn, Twitter, and Instagram.

The survey was created in Arabic language and participation was encouraged without incentives. Response to all items was mandatory for successful submission of the survey form. Pilot testing was deemed unnecessary based on the previous validation of the survey instruments in Arabic [67].

The introductory section of the survey clearly listed eligibility criteria for participation as follows: (1) Being either a nurse of physician; (2) Currently working as a health professional in Jordan; (3) Aged 18 years or older. It was also clearly indicated that participation was completely anonymous and voluntary with full confidentiality of the collected data. Elective participation was ensured by the following introductory mandatory item “Do you agree to participate in this study?”, with immediate closure of the survey link in case of selection of “No” as the answer.

This study was approved by the Scientific Research Committee at the School of Medicine—University of Jordan (Reference No. 206/2023/67) and by the Institutional Review Board at Jordan University Hospital (IRB-JUH, decision number: 44/2023, reference number: 10/2023/4564).

### 2.2. Survey Instrument

The survey instrument was adopted from previous studies with a slight modification in the context of Mpox vaccination [21,39,44,67]. The questionnaire comprised six sections as follows:

#### 2.2.1. Sociodemographic Variables

The sociodemographic variables assessed in the current study included:

- Age as a scale ranging from 18 to 75 years, and later dichotomized based on the study sample median into two categories (≤32 years vs. >32 years);
- Sex as two categories (male vs. female);
- The highest educational level attained as two categories: (1) undergraduate including diploma, and Bachelor of Science (BSc) degrees; (2) postgraduate including Master in Science (MSc), higher specialization, fellowship and Doctor of Philosophy (PhD) degrees;
- Marital status as two categories: (1) married; (2) single, widow/widower and divorced;
- Subjective assessment of the current financial status as two categories: (1) poor or fair; (2) good or excellent;
- Current place of residence as two categories: (1) the Capital, Amman; (2) Outside the Capital;
- Occupational category (nurse vs. physician);
- Seniority level as two categories: (1) Less than 10 years of work experience; (2) 10 years or more of work experience;
- Nationality as two categories (Jordanian vs. non-Jordanian); and
- Self-reported history of chronic disease (e.g., hypertension (HTN), diabetes mellitus (DM), asthma, cardiovascular disease (CVD)), as two categories (yes vs. no).

#### 2.2.2. Knowledge of Mpox

Assessment of Mpox knowledge prior to the current study was evaluated as follows [41,44,65]. An initial question was asked to determine if the participant had heard of Mpox prior to the study as follows: “Have you heard of Mpox prior to this study?” with “yes” vs. “no” as possible answers. Assessment of Mpox knowledge using the subsequent ten questions was only undertaken for the participants who answered “yes”.

There were three possible responses to the following ten knowledge items: “yes” vs. “no” vs. “I do not know”:

- Mpox is caused by bacteria (incorrect);
- There is a global outbreak of Mpox (correct);
- Mpox is endemic in Western and Central Africa (correct);
- Skin rash is a symptom of Mpox (correct);
- Mpox and smallpox symptoms are similar (correct);
- Mpox is easily transmitted in humans (incorrect);
- Mpox is spreading among male homosexuals to a large extent (correct);
- Mpox virus does not infect children or females (incorrect);
- Mpox can be treated with antibiotics (incorrect); and
- Currently, vaccination is available to prevent Mpox (correct).

#### 2.2.3. Vaccination Behavior

Previous behavior towards vaccination among the study respondents was assessed using the following items:

- Have you received the first two doses of COVID-19 vaccination?
- Have you received the third booster dose of COVID-19 vaccination?
- Have you received influenza vaccination this winter season or during the previous winter season? And
- Have you ever received the influenza vaccine in the years prior to the COVID-19 pandemic?

#### 2.2.4. Intention to Receive Mpox Vaccination

The assessment of the willingness to get Mpox vaccination among the study respondents was conducted using the following item, with three possible responses (yes, maybe, no):

- If a safe and effective Mpox vaccine is available free of charge, would you be willing to receive it?

#### 2.2.5. Psychological Determinants of Mpox Vaccination

The psychological determinants of Mpox vaccination were evaluated using nine items, each scored on a 5-point Likert scale (strongly disagree, disagree, neutral/no opinion, agree and strongly agree) [20,21,67]:

- I will receive Mpox vaccination if it is effective;
- I will receive Mpox vaccination if the Ministry of Health recommends the vaccine;
- It is important that I get Mpox vaccination to protect community members with weaker immunity;
- I will not receive Mpox vaccination because I have a strong immune system which will protect me from the disease;
- I will not receive Mpox vaccination because the disease is not dangerous;
- I would not receive Mpox vaccination if I had to pay for the vaccine;
- I will not receive Mpox vaccination if I have to register on online platforms or wait for a long time;
- Before receiving the vaccine, it is important that I weigh the benefits and potential harm of the vaccine; and
- It is important that I fully understand all about Mpox vaccination before I decide to receive it.

#### 2.2.6. Attitude towards Compulsory COVID-19, Influenza, and Mpox Vaccination

The attitude towards compulsory vaccination among the general public and among HCWs was assessed using six items, each scored on a 5-point Likert scale (strongly disagree, disagree, neutral/no opinion, agree and strongly agree):

- COVID-19 vaccination should be compulsory for all members of society;
- COVID-19 vaccination should be compulsory for all HCWs;
- Influenza vaccination should be compulsory for all members of society;
- Influenza vaccination should be compulsory for all HCWs;
- Mpox vaccination should be compulsory for all members of society; and
- Mpox vaccination should be compulsory for all HCWs.

### 2.3. Study Measures

#### 2.3.1. Willingness to Get Mpox Vaccination

The primary study measure was the intention to get Mpox vaccination if it was safe and provided free of charge with the following classification:

- Participants who answered “yes” were considered as the vaccine acceptance group;
- Participants who answered “maybe” were considered as the vaccine hesitancy group; and
- Participants who answered “no” were considered as the vaccine rejection group.

#### 2.3.2. Mpox Knowledge Score (Mpox K Score)

Assessment of Mpox knowledge was performed using the Mpox K score which comprised the total score of the ten knowledge items among the participants who had heard of Mpox prior to the study. For each item, the correct response was scored as “2”, “I do not know” was scored as “1” and incorrect responses were scored as “zero”. This yielded a potential minimum Mpox K score of zero and a maximum score of 20. Mpox K score was dichotomized based on the median in the study sample, to form two groups:

- Participants with Mpox K score ≤12 were allocated to a “lower Mpox K score” group; and
- Participants with Mpox K score >12 were allocated to a “higher Mpox K score” group.

#### 2.3.3. Previous Vaccination Behavior Score (VB Score)

Assessment of previous vaccination behavior was conducted using four items that inquired about the previous history of primary COVID-19 vaccination uptake, booster COVID-19 vaccine uptake, influenza vaccine uptake in the last two winter seasons and influenza vaccine uptake prior to COVID-19. For each item, the self-reported vaccine uptake was scored as “1”, while the “no” response was scored as “zero”. The resultant VB score had a potential minimum of zero and a maximum score of 4. The VB score was dichotomized based on the median in the study sample, to form two groups:

- Participants with VB score ≤2 were allocated to a “lower VB score” group, indicating less active vaccine uptake behavior; and
- Participants with VB score >2 were allocated to “higher VB score” group, indicating more active vaccine uptake behavior.

#### 2.3.4. 5C Scale for Psychological Determinants of Mpox Vaccination (5C Scale)

The 5C psychological determinants of vaccination (Confidence, Collective responsibility, Constraints, Calculation, and Complacency) were assessed as follows. For each item, a 5-point Likert scale was used with the strongly agree response scored as “1”, the agree response scored as “2”, neutral/no opinion response scored as “3”, the disagree response scored as “4”, and the strongly disagree response scored as “5”. The scoring was reversed for the confidence and collective responsibility items. The resultant 5C scale had a potential minimum of nine and a maximum score of 45. An acceptable internal consistency of the 5C scale was ensured by a Cranach’s α value of 0.706. The 5C scale score was dichotomized based on the median in the study sample, to form two groups:

- Participants with 5C scale ≤ 26 were allocated to a “lower 5C scale” group; and
- Participants with 5C scale > 26 were allocated to a “higher 5C scale” group.

#### 2.3.5. Attitude Scale towards Compulsory Vaccination

The attitude towards compulsory vaccination was assessed using six items. For each item, a 5-point Likert scale was used with the strongly agree response scored as “5”, the agree response scored as “4”, the neutral/no opinion response scored as “3”, the disagree response scored as “2”, and the strongly disagree response scored as “1”. The resultant scale had a potential minimum score of six and a maximum score of 30. The excellent internal consistency of the scale was ensured by a Cranach’s α value of 0.935. The score was dichotomized based on the median in the study sample, to form two groups:

- Participants with a score ≤18 were considered as having a “lower compulsory vaccination scale” attitude, denoting a neutral or less favorable attitude towards compulsory vaccination; and
- Participants with a score >18 were considered as having a “higher compulsory vaccination scale” score, denoting a favorable attitude towards compulsory vaccination.

### 2.4. Statistical Analysis

Analyses were performed using IBM SPSS Statistics for Windows, Version 26.0. Armonk, NY, USA: IBM Corp. Univariate analyses were conducted using the chi-squared test for categorical variables and the Mann–Whitney *U* test (M-W) or Kruskal–Wallis test (K-W), for dichotomous or trichotomous variables in comparison with scale variables. For scale variables, normality was checked using the Kolmogorov–Smirnov test (K-S).

Multivariate analyses were conducted following univariate analysis with variables showing *p* < 0.100. Multicollinearity in multinomial logistic regression was checked by inspecting the variance inflation factor (VIF) values, with VIF values >5 indicating severe correlation between the predictor variable and other variables in the model. The statistical significance level was *p* < 0.050.

## 3. Results

### 3.1. Study Sample

The final study sample comprised 495 respondents, with 302 nurses (61.0%) and 193 physicians (39.0%). A summary of the characteristics of the sample, stratified by occupational category, is shown in Table 1.

### 3.2. Unsatisfactory Level of Mpox Knowledge in the Study Sample

In the whole study sample, 430 participants (86.9%) indicated that they had heard of Mpox prior to the study. For these participants, the responses to each knowledge item from nurses and physicians are shown in (Table 2).

**Figure 1:**
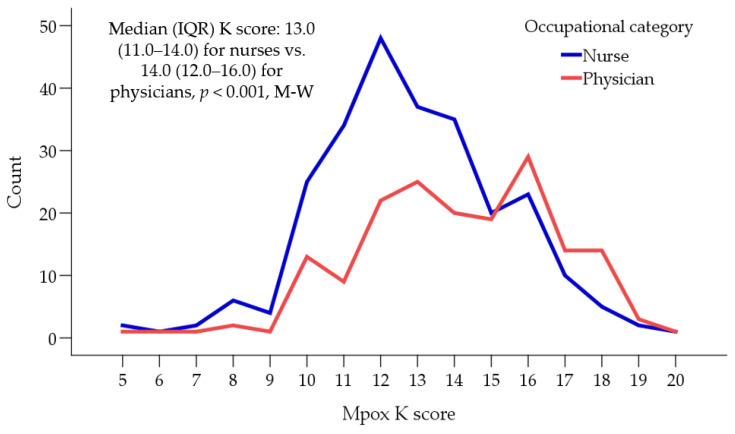
The distribution of monkeypox (Mpox) knowledge score per occupational category. IQR: Interquartile range, M-W: Mann–Whitney *U* test. Mpox K score: Monkeypox knowledge score as a scale variable.

Among the participants with a valid Mpox K score (*n* = 430), the mean K score was 13.3 ± 2.7 (median = 13.0, IQR: 12.0–15.0). A higher mean Mpox K score was observed among physicians compared to nurses (14.0 ± 2.7 vs. 12.8 ± 2.5, *p* < 0.001, M-W, Figure 1).

The Mpox K score analysis, dichotomized into higher and lower knowledge categories, revealed that higher K scores with a statistically significant difference were observed among physicians and participants with postgraduate educational level (Table 3).

Multivariate analysis revealed that male sex (odds ratio (OR): 1.7, 95% confidence interval (CI): 1.1–2.6, *p* = 0.012), and occupation as a physician (OR: 2.0, 95% CI: 1.3–3.0, *p* = 0.002) were significantly correlated with higher Mpox K score, while educational level did not show a statistically significant difference (*p* = 0.122).

### 3.3. Previous Vaccination Behavior in the Study Sample

**Figure 2:**
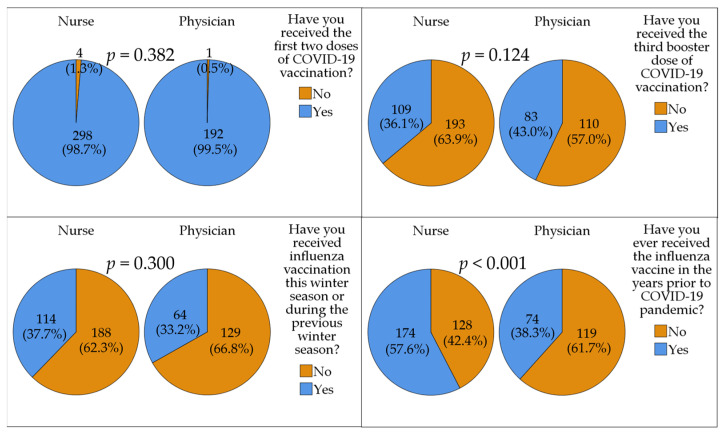
The previous uptake of COVID-19 and influenza vaccines in the study sample per occupational category. *p* values were calculated using chi-squared test. COVID-19: Coronavirus disease 2019.

The previous uptake of COVID-19 and influenza vaccines in the study sample is shown in (Figure 2).

Higher uptake of influenza vaccine in the seasons prior to COVID-19 was seen among participants older than 32 years, among married participants, among participants with a seniority level of more than 10 years, and among nurses. Advanced seniority level was also significantly associated with the uptake of influenza vaccination following COVID-19. Residence in Amman was associated with a higher uptake of booster COVID-19 vaccine doses and with influenza vaccine uptake during COVID-19 and this association was statistically significant (Table 4).

### 3.4. A Low Rate of Willingness to Get Mpox Vaccination Was Found in the Study Sample

In the whole study sample, the intention to receive Mpox vaccination was reported by 143 participants (28.9%), while hesitancy was reported among 165 participants (33.3%). Resistance to Mpox vaccination was found among 187 participants (37.8%).

The association of intention to get Mpox vaccination with different study variables is shown in Table 5.

### 3.5. Psychological Predictors for Willingness to Receive Mpox Vaccination in the Study Sample

**Figure 3:**
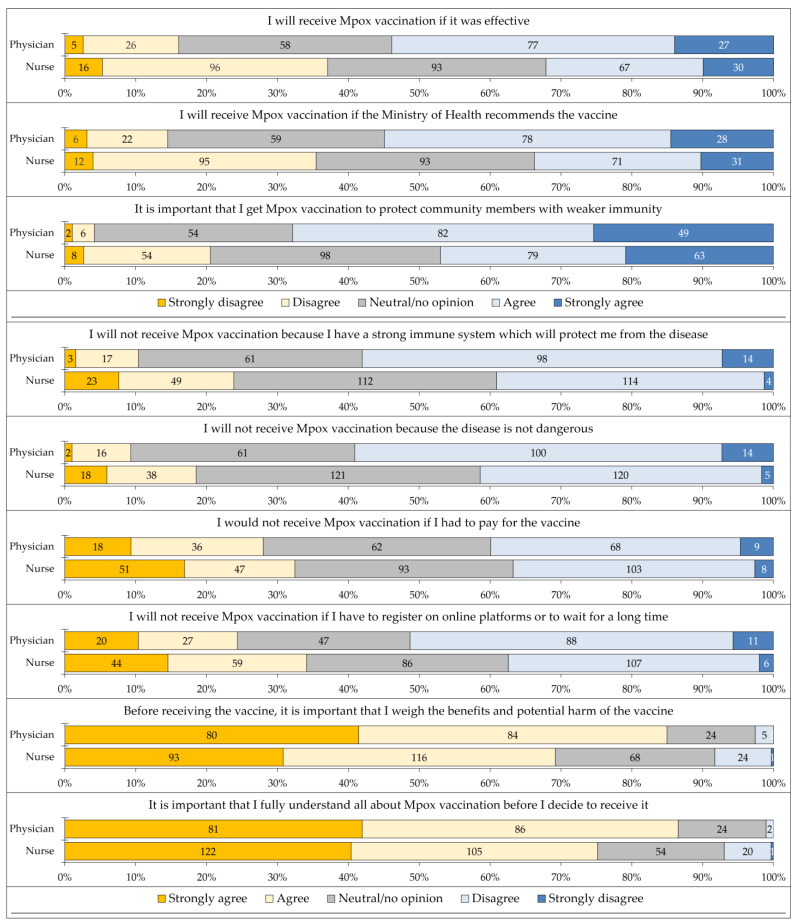
The responses to the nine items that assessed the 5C psychological predictors of vaccine acceptance stratified by occupation. Mpox: Monkeypox.

The responses to the nine items that assessed the 5C psychological predictors of vaccine acceptance stratified by occupation are shown in Figure 3.

The associations of different study variables with each of the 5C scale items assessed as a scale variable are shown in Table 6.

For confidence and collective responsibility items, significantly higher mean values indicating higher confidence and collective responsibility were seen among younger participants, single/divorced/widows/widowers, participants residing in Amman, physicians, and participants with less than 10-year working experience (Table 6).

For complacency items, significantly higher mean values indicating lower complacency towards Mpox were seen among younger participants, females, single/divorced/widows/widowers, participants with good/excellent self-reported financial status, participants residing in Amman, physicians, and participants with less than 10-year working experience (Table 6). For constraints items, significantly higher mean values indicating lower constraints perceived towards Mpox vaccination were seen among younger participants, females, single/divorced/widows/widowers, participants residing in Amman, and physicians (Table 6). For calculation items, significantly higher mean values indicating lower calculation were seen among older participants, females, married participants, participants with ≥10-year working experience, and participants with a history of chronic disease (Table 6).

Higher overall 5C mean scores indicating lower psychological barriers towards Mpox vaccination were observed among younger participants, single/divorced/widows/widowers, participants residing in Amman, physicians, and participants with less than 10-year working experience (Table 6). Stratified by Mpox vaccine acceptance group, the responses to each of the 5C items are shown in Table 7.

### 3.6. Multivariate Analysis of the Factors Associated with Mpox Vaccine Acceptance

Multivariate analysis was conducted to determine the variables significantly correlated with higher intention to get Mpox vaccination. Psychological determinants of Mpox vaccination were significantly associated with higher intention to receive the vaccine among the acceptance group compared to the hesitancy and resistant groups. Higher hesitancy/resistance to vaccination was also noticed among the participants with lower scores on vaccine uptake (Table 8).

### 3.7. Attitude of the Participants towards Compulsory Vaccination

The overall attitude towards compulsory vaccination, divided by occupational category, is shown in Table 9.

**Figure 4:**
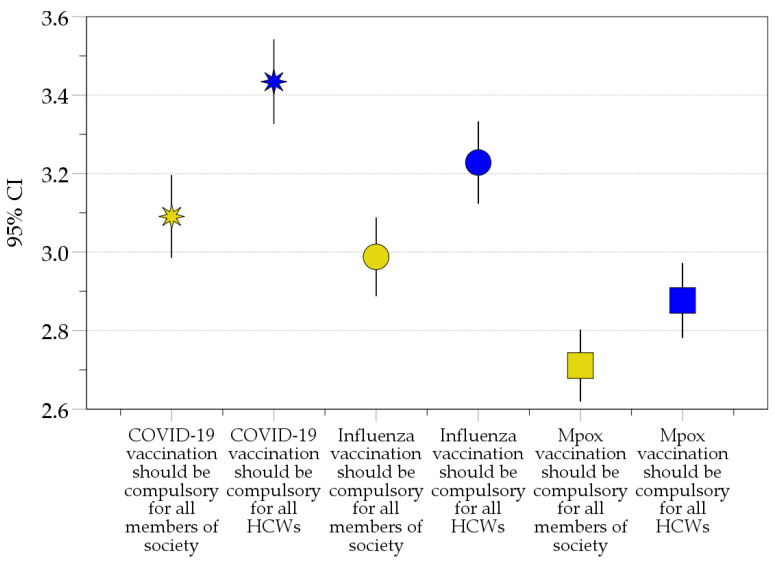
The mean values for responses to compulsory vaccination items. CI: confidence interval of the mean; COVID-19: COVID-19: Coronavirus disease 2019; HCWs: Healthcare workers; Mpox: Monkeypox. The items involving vaccination among the general public are represented in yellow, while the items involving vaccination of HCWs are represented in blue. COVID-19 vaccination is represented by eight-pointed stars, influenza vaccination is represented by circles, and Mpox vaccination is represented by squares.

Higher mean scores were observed for compulsory vaccination of HCWs compared to compulsory vaccination of the general public for COVID-19 vaccination (mean: 3.4 ± 1.2 vs. 3.1 ± 1.2, *p* < 0.001, M-W), for influenza vaccination (mean: 3.2 ± 1.2 vs. 3.0 ± 1.1, *p* = 0.001, M-W), and for Mpox vaccination (mean: 2.9 ± 1.1 vs. 2.7 ± 1.0, *p* = 0.015, M-W). Higher compulsory vaccination mean scores were also noticed for COVID-19 vaccination compared to influenza and Mpox vaccination (mean: 3.3 ± 1.2 vs. 3.1 ± 1.2 vs. 2.8 ± 1.1, *p* < 0.001, K-W, Figure 4).

The mean compulsory vaccination score in the whole study sample was 18.3 ± 6.0 (median = 18.0, IQR: 13.0–23.0). Higher compulsory vaccination scores indicating more agreement towards compulsory vaccination was seen among the participants with a previous history of vaccine uptake and among the participants with higher 5C scores (Table 10).

Multivariate analysis showed that agreement with compulsory vaccination was associated with a previous history of vaccine uptake reflected in higher VB scores and with higher 5C scores as well (Table 11).

## 4. Discussion

The decision to get vaccinated is a complex multifactorial process. The current study pointed to psychological factors and past vaccination behavior as the main determinants of the willingness to get Mpox vaccination in a sample of nurses and physicians practicing in Jordan.

Indeed, previous studies in the context of different vaccine types and different populations showed the significant role of the psychological factors in the decision-making process that vaccine uptake entails [20,68,69,70,71,72]. However, our study findings suggested that psychological factors may play a more significant role in shaping individuals’ decisions to get vaccinated compared to socio-demographic variables (e.g., occupational category, sex, age, financial status), which have been previously considered as important determinants of the intention to get vaccinated [11,73].

Additionally, past vaccination behavior—as indicated by the history of vaccine uptake for different types of vaccines—can influence the prospects of receiving vaccination in the future [74,75,76,77]. The higher likelihood of vaccine acceptance among individuals with previous vaccination history may be attributed to their positive experience in terms of benefits gained from vaccination, coupled with the minimal associated risks [78].

The major result of this study was the finding of a low intention to get Mpox vaccination among the surveyed HCWs in Jordan. Specifically, 38% of those surveyed exhibited absolute resistance to safe, effective, and free-of-charge Mpox vaccine. Additionally, 33% of the surveyed HCWs responded with “maybe” to the same survey item suggesting hesitancy to Mpox vaccination.

In this study, the finding of an Mpox vaccine acceptance rate of only 29% is lower compared to the pooled estimate in a recent meta-analysis that involved HCWs surveyed in four different studies showing an acceptance rate of 63% [43,56,79,80,81]. Additionally, a recent review by Lounis and Riad pointed to the issue of possible Mpox vaccination hesitancy among health professionals despite the higher rates of vaccine acceptance compared to the rates reported among the general public worldwide [57].

From a wider perspective, generally low levels of intentions to get Mpox vaccination were reported among the general public, university students and health professionals worldwide [56,57]. This may be attributable to the perception of disease risks being concentrated among certain risk groups (e.g., MSM) [82]. Specifically, a recent survey among adults in the U.S. showed low knowledge levels and vaccination intentions at 46% level if the vaccine is recommended [83]. A recent study that assessed the knowledge, attitudes, and willingness to vaccinate against Mpox among Pakistani university students reported a willingness to receive Mpox vaccination at a higher level of 68%, with 35% willing to pay for the vaccine [84]. Among a sample of a most-at-risk group (MSM) in France, Mpox vaccination hesitancy was reported at a rate of 34% despite the positive attitude towards vaccination [85]. Another recent study among medical workers in China reported that a majority of participants (65%) supported the promotion of Mpox vaccination, particularly among health practitioners and immunodeficient populations [86]. Another study among HCWs in China showed high willingness to get Mpox vaccination at a rate of 90% [87]. An early study that was conducted among Italian medical professionals showed 59% in favor of using variola vaccine to prevent Mpox, emphasizing the need for information campaigns for first-line medical responders [43]. A later study among 111 HCWs in Algeria by Lounis et al. showed that only 39% of the participants were in favor of Mpox vaccination [88]. A very low rate of Mpox vaccine acceptance (9%) was reported among HCWs in the Czech Republic [89].

The high prevalence of reluctance to get Mpox vaccination as reported in this study can be related to the following possible factors. First, complacency towards the disease is understandable in Jordan among other Middle East countries considering the limited number of Mpox cases reported in the region [36]. This is related to the unequal distribution of Mpox cases; historically, the disease affected Central and West Africa, while the recent 2022 outbreak involved cases mainly in the U.S. and Europe [29,35,36]. Therefore, the perceived threat from the disease might be low among health professionals in Jordan, with a number of them lacking knowledge of the disease altogether [44]. Despite that, the levels of complacency towards Mpox in this study were relatively low as indicated by the two 5C items, which suggests that the low perceived threat might be linked to the extremely low number of cases reported in the Middle East with a single case in Jordan, rather than low perceived risk from the disease itself [36]. Second, the context of Mpox vaccination intention assessment in terms of place and time should be taken into account. The current study was conducted following the COVID-19 pandemic, which was accompanied by high rates of vaccination hesitancy linked to misinformation and various conspiratorial ideas that were widely prevalent in Jordan, even among health professionals [39,44,45,65,90]. Thus, a spillover of these conspiratorial ideas into the Mpox outbreak is an expected outcome and should be further investigated, considering its potential link with negative health-seeking behavior. Indeed, conspiracy ideas in the Arab region started to circulate shortly after the reporting of the Mpox outbreak [91,92].

This study identified the critical role of psychological predictors in shaping the intentions to receive a safe and effective vaccine, which can serve as a model to understand the factors that should be considered for vaccination against emerging infections. Further dissection of these psychological factors revealed significantly higher levels of confidence and collective responsibility among the Mpox vaccine acceptance group, with agreement ranging between 84% and 90% for these items. On the other hand, confidence and collective responsibility levels were significantly lower among the Mpox vaccine hesitancy group (range of agreement 37–59%), and much lower among the vaccine resistance group (6% to 29% level of agreement). Prior evidence highlighted the value of elaboration on vaccine safety and efficacy in the communication strategies aiming to promote vaccination [93,94]. Additionally, previous studies in the context of different types of vaccines, including those for COVID-19, seasonal influenza, and human papillomavirus (HPV), have highlighted the importance of vaccine confidence, including trust in health institutions and professionals as a valuable tool to address vaccination hesitancy [95,96,97]. Furthermore, the role of collective responsibility appears to be of notable importance among health professionals, serving as a driving factor for vaccine acceptance [98]. Certain categories, particularly those in direct contact with immunodeficient individuals and those at risk, should be particularly encouraged as regards their role in community protection [99].

In addition to the lower levels of confidence and collective responsibility, higher levels of complacency and perceived constraints were associated with Mpox vaccination resistance/hesitancy in this study. Specifically, the agreement with the complacency items was noticed at levels ranging between 27% to 33% in the resistance group as opposed to only 6% to 11% among the hesitancy and acceptance groups. Complacency is an important determinant of weak vaccine acceptance and it is defined as a limited perceived threat from the disease and the feeling that the immune system can protect one from dangers [24]. Overall, the levels of complacency were low in the whole study sample which could be linked to recording of severe cases and mortalities as a result of Mpox, as well as the grave consequences of its better known and closely related disease, namely smallpox [26,100].

The results of this study also pointed to the significant link between the high levels of perceived constraints and resistance to Mpox vaccination. Therefore, to address this issue, it is important to improve convenience in the vaccination experience by reducing these constraints. This can be achieved, for example, by the provision of free vaccination [101]. Lowering constraints should be a key objective in promoting vaccination strategies among HCWs, especially in the event of an emerging infection that requires immediate vaccination of these key groups [15,102].

High levels of calculation in terms of weighing the risks and benefits from vaccination as well as the importance of getting sufficient information about the vaccine were seen across the three vaccine attitude groups in this study. However, higher levels of calculation were seen among the vaccine acceptance group (84–87%), compared to the hesitancy group (78–79%), and resistance group (67–74%). Thus, a special emphasis should be put into highlighting the benefits of vaccination as opposed to the minimal risks of the vaccines, particularly among HCWs at high risk of virus acquisition [103,104].

The fine granularity of the psychological factors in the context of Mpox vaccination has been recently investigated among Nigerian HCWs, with low levels of confidence and collective responsibility as well as high levels of constraints and complacency hindering vaccine acceptance [105]. Additionally, the central role of these psychological determinants of vaccination, as modeled through the 5C scale, has also been highlighted in the context of COVID-19 and influenza vaccine acceptance among Kuwaiti and Jordanian HCWs [68,69].

A variability in Mpox vaccine acceptance was detected in this study based on different socio-demographic characteristics, with higher vaccine acceptance among the participants who were younger, single, residents in Amman, physicians and those with less years of work experience. In spite of this, multivariate analysis failed to show significant statistical differences, indicating a confounding effect. Additionally, multivariate analysis only indicated the relevance of psychological factors and past vaccination history as factors associated with higher Mpox vaccine acceptance. This result is in line with previous evidence showing variability in results regarding the role of socio-demographics or occupation as determinants of vaccine acceptance, which may be the result of different study designs including sampling issues [25,106,107].

In contrast to the previous studies that warned of the low level of knowledge regarding Mpox and its subsequent negative effect on outbreak response in terms of awareness of risks and preventive measures, our results did not show a statistically significant impact of Mpox knowledge on the intention to receive the vaccine [57,89]. Previous studies showed that unsatisfactory Mpox knowledge is commonplace among health professionals [41,43,89,108]. For example, Gonzales-Zamora et al. showed that only 61% of a sample comprising Peruvian physicians were aware that FDA-approved Mpox vaccines are available [109]. Similarly, Sahin et al. showed a low level of Mpox knowledge among Turkish physicians, with only 31% planning to get Mpox vaccination [110]. This can be understood based on the previous concentration of Mpox cases in endemic regions with minimal exposure outside Africa [35].

The concept of previous vaccination behavior as an important determinant of vaccine acceptance has been previously illustrated [76,77]. Despite the limitations of our approach in assessing past vaccination behavior which relied solely on two vaccines (COVID-19 and influenza), the significant differences observed among the three Mpox vaccine attitude groups indicate the relevance of past vaccination history as a determinant of the intention to get vaccinated. Thus, the strategies to avert vaccination resistance/hesitancy can benefit from a special focus on the individuals with a history of low vaccine uptake for different vaccine types. However, achieving this goal is not a straightforward task based on the previous evidence that vaccine resistance can be hardwired and thus, it might be wise to prioritize the fence sitters (hesitant individuals) for such strategies, considering the higher likelihood of such a group responding to intervention [111,112].

Finally, we investigated the attitude of the participants towards compulsory vaccination in light of the divided opinion regarding such a strategy [113,114,115,116]. Although evidence exists showing that vaccine mandates, particularly for childhood vaccination, are linked with increased vaccine coverage, careful assessment of the potential risks of this strategy is also needed [117]. Imposing mandates on vaccination implies sacrificing some personal freedom with the subsequent perceived threat of losing a valued behavior [118,119]. Consistent with the previous evidence, the divided opinion regarding compulsory vaccination was reflected in our study sample with variability depending on the type of vaccine. The highest level of agreement towards compulsory vaccination was seen among physicians towards COVID-19 vaccination of HCWs while the lowest level was seen among nurses towards Mpox vaccination for the general public.

The gradual decrease in the prevalence of support for compulsory vaccination with the highest support being for COVID-19 vaccination followed by influenza then Mpox can be linked to the perceived threat, since the feared consequences of COVID-19 were recognizable to a large degree especially among HCWs who represented the frontline first responders during the pandemic [120]. In contrast, the fear of Mpox was not as discernible in the study sample as inferred through the lowest percentage of being in favor of mandatory Mpox vaccination, likely related to the low number of cases reported in the region.

The intricate details of varying attitudes towards compulsory vaccination based on the type of vaccine and population of interest as reported in this study require special attention. This comes in light of the previous evidence that vaccine mandates might backfire because of the divided opinion regarding such a strategy among the general public as well as the HCWs who can be advocates for such a strategy [121,122]. In all cases, mandatory vaccination can be considered carefully as a last resort following the implementation of educational campaigns highlighting the benefits of vaccination coupled with emphasis on vaccine efficacy and safety [123].

The correlation of psychological factors with attitudes towards compulsory vaccination was revealed in multivariate analysis. Additionally, a respondent’s history of vaccine uptake was associated with a favorable attitude towards mandatory vaccination. Thus, the implementation of vaccine mandates should consider these factors, which can help in the success of this strategy. The absence of association between the socio-demographic variables and attitude to compulsory vaccination can point to the uniform existence of divided opinion towards vaccine mandates.

Despite the valuable results inferred from the findings of the current study, we admit that there are several limitations that could compromise the generalizability of our results. First, vaccination hesitancy is a context-, time- and place-specific phenomenon which means that the results can only be used as a guide to the factors that need to be further investigated, ideally with follow-up longitudinal studies. Second, selection bias should be considered, based on the sampling approach; hence, future studies should consider stratified random sampling. Third, the convenience sampling approach is also prone to motivation bias with the possibility that some HCWs participated in the study to express specific opinions regarding compulsory vaccination. Fourth, despite the use of a survey instrument validated in Arabic which helped to waive the pilot and validation steps needed in survey studies, measurement bias cannot be ruled out. Fifth, the potential for variability in responses based on the specific phrasing of the survey items assessing vaccine acceptance should be considered as well. Finally, the assessment of attitudes towards compulsory vaccination entails ethical perspectives which should be investigated in future studies since this issue was out of the scope of the current study.

## 5. Conclusions

In this study, we took the opportunity of the global Mpox outbreak that was reported in May 2022 as a model to study the factors linked with Mpox vaccine hesitancy or rejection. The findings suggested the central role of psychological factors as significant determinants for the willingness to get vaccinated against Mpox. Specifically, if a vaccination campaign is needed to address a re-emerging infectious disease, facilitating personal calculations of the correct decision in terms of vaccine uptake can be of prime importance. This can be supported by highlighting the benefits and importance of vaccination and showing the minimal risks of vaccination. Additionally, our results highlighted the need to prioritize reaching health professionals who did not receive vaccines before, despite the difficulty of achieving such an aim.

Regarding the implementation of compulsory vaccination, the findings of this study showed the need to consider each vaccine type and each population separately. Additionally, the consideration of psychological factors can help to achieve the success of this strategy.

## Figures and Tables

**Table 1:** Sample characteristics stratified per occupational category (N = 495).

Variable	Category	Occupational Category	
		Nurse N ^2^ (%)	Physician N (%)
Age	≤32 years	78 (25.8)	167 (86.5)
	>32 years	224 (74.2)	26 (13.5)
Sex	Male	107 (35.4)	97 (50.3)
	Female	195 (64.6)	96 (49.7)
Highest educational level	Undergraduate	283 (93.7)	149 (77.2)
	Postgraduate	19 (6.3)	44 (22.8)
Marital status	Single, divorced, widow/widower	62 (20.5)	137 (71.0)
	Married	240 (79.5)	56 (29.0)
Self-reported financial status	Poor or fair	163 (54.0)	65 (33.7)
	Good or excellent	139 (46.0)	128 (66.3)
Residence	Amman	143 (47.4)	141 (73.1)
	Outside the Capital	159 (52.6)	52 (26.9)
Seniority level	1–9 years	109 (36.1)	176 (91.2)
	10 years or more	193 (63.9)	17 (8.8)
Nationality	Jordanian	299 (99.0)	179 (92.7)
	Non-Jordanian	3 (1.0)	14 (7.3)
Self-reported history of chronic disease (e.g., HTN, DM, asthma, CVD) ^1^	Yes	41 (13.6)	10 (5.2)
	No	261 (86.4)	183 (94.8)

^1^ HTN: hypertension; DM: diabetes mellitus; CVD: cardiovascular disease; ^2^ N: Number.

**Table 2:** Mpox knowledge per item divided by occupational category.

Mpox ^1^ Knowledge Item	Response	Occupational Category		*p* Value ^3^
		Nurse N ^2^ (%)	Physician N (%)	
Have you heard of Mpox prior to this study?	Yes	255 (84.4)	175 (90.7)	**0.045**
	No	47 (15.6)	18 (9.3)	
Mpox is caused by bacteria	Correct	103 (40.4)	125 (71.4)	**<0.001**
	I do not know	75 (29.4)	32 (18.3)	
	Incorrect	77 (30.2)	18 (10.3)	
There is a global outbreak of Mpox	Correct	118 (46.3)	58 (33.1)	**0.022**
	I do not know	43 (16.9)	40 (22.9)	
	Incorrect	94 (36.9)	77 (44.0)	
Mpox is endemic in Western and Central Africa	Correct	139 (54.5)	75 (42.9)	**0.034**
	I do not know	87 (34.1)	81 (46.3)	
	Incorrect	29 (11.4)	19 (10.9)	
Skin rash is a symptom of Mpox	Correct	227 (89.0)	155 (88.6)	0.125
	I do not know	18 (7.1)	18 (10.3)	
	Incorrect	10 (3.9)	2 (1.1)	
Mpox and smallpox symptoms are similar	Correct	193 (75.7)	127 (72.6)	**0.021**
	I do not know	31 (12.2)	36 (20.6)	
	Incorrect	31 (12.2)	12 (6.9)	
Mpox is easily transmitted in humans	Correct	86 (33.7)	61 (34.9)	**0.017**
	I do not know	63 (24.7)	62 (35.4)	
	Incorrect	106 (41.6)	52 (29.7)	
Mpox is spreading among male homosexuals to a large extent	Correct	110 (43.1)	105 (60.0)	**0.002**
	I do not know	121 (47.5)	62 (35.4)	
	Incorrect	24 (9.4)	8 (4.6)	
Mpox virus does not infect children or females	Correct	145 (56.9)	113 (64.6)	0.054
	I do not know	85 (33.3)	55 (31.4)	
	Incorrect	25 (9.8)	7 (4.0)	
Mpox can be treated with antibiotics	Correct	81 (31.8)	106 (60.6)	**<0.001**
	I do not know	95 (37.3)	62 (35.4)	
	Incorrect	79 (31.0)	7 (4.0)	
Currently, vaccination is available to prevent Mpox	Correct	65 (25.5)	32 (18.3)	0.165
	I do not know	119 (46.7)	95 (54.3)	
	Incorrect	71 (27.8)	48 (27.4)	

^1^ Mpox: Monkeypox; ^2^ N: Number; ^3^ *p* value: Calculated using chi-squared test. Statistically significant *p* values are highlighted in bold style.

**Table 3:** Factors related with Mpox knowledge among the study respondents.

Variable	Category	Mpox K Score ^1^		*p* Value ^2^
		≤12 N ^2^ (%)	>12 N (%)	
Age	≤32 years	86 (39.3)	133 (60.7)	0.753
	>32 years	86 (40.8)	125 (59.2)	
Sex	Male	55 (31.6)	119 (68.4)	**0.003**
	Female	117 (45.7)	139 (54.3)	
Highest educational level	Undergraduate	157 (42.0)	217 (58.0)	**0.030**
	Postgraduate	15 (26.8)	41 (73.2)	
Marital status	Single, divorced, widow/widower	77 (41.6)	108 (58.4)	0.551
	Married	95 (38.8)	150 (61.2)	
Self-reported financial status	Poor or fair	79 (40.9)	114 (59.1)	0.722
	Good or excellent	93 (39.2)	144 (60.8)	
Residence	Amman	94 (37.2)	159 (62.8)	0.150
	Outside the Capital	78 (44.1)	99 (55.9)	
Occupational category	Nurse	122 (47.8)	133 (52.2)	**<0.001**
	Physician	50 (28.6)	125 (71.4)	
Seniority level	1–9 years	97 (38.3)	156 (61.7)	0.401
	10 years or more	75 (42.4)	102 (57.6)	
Nationality	Jordanian	163 (39.5)	250 (60.5)	0.266
	Non-Jordanian	9 (52.9)	8 (47.1)	
Self-reported history of chronic disease	Yes	18 (41.9)	25 (58.1)	0.793
	No	154 (39.8)	233 (60.2)	

^1^ Mpox K score: Monkeypox knowledge score calculated based on 10 knowledge items. Please notice that this score was only calculated for the participants who had heard of Mpox prior to the study; therefore, the number of respondents with a valid Mpox K score will not add up to the total number of study respondents. ^2^ *p* value: Calculated using chi-squared test. Statistically significant *p* values are highlighted in bold style.

**Table 4:** Detailed self-reported vaccination history among the study participants.

Variable	Category	Have You Received the First Two Doses of COVID-19 ^2^ Vaccination?			Have You Received the Third Booster Dose of COVID-19 Vaccination?			Have You Received Influenza Vaccination This Winter Season or during the Previous Winter Season?			Have You Ever Received the Influenza Vaccine in the Years Prior to COVID-19 Pandemic?		
		No	Yes	*p* Value ^3^	No	Yes	*p* Value	No	Yes	*p* Value	No	Yes	*p* Value
Age	≤32 years	4 (1.6)	241 (98.4)	0.170	155 (63.3)	90 (36.7)	0.353	162 (66.1)	83 (33.9)	0.339	148 (60.4)	97 (39.6)	**<0.001**
	>32 years	1 (0.4)	249 (99.6)		148 (59.2)	102 (40.8)		155 (62.0)	95 (38.0)		99 (39.6)	151 (60.4)	
Sex	Male	0	204 (100)	0.060	115 (56.4)	89 (43.6)	0.064	132 (64.7)	72 (35.3)	0.796	98 (48.0)	106 (52.0)	0.488
	Female	5 (1.7)	286 (98.3)		188 (64.6)	103 (35.4)		185 (63.6)	106 (36.4)		149 (51.2)	142 (48.8)	
Highest educational level	Undergraduate	4 (0.9)	428 (99.1)	0.624	265 (61.3)	167 (38.7)	0.876	278 (64.4)	154 (35.6)	0.705	218 (50.5)	214 (49.5)	0.511
	Postgraduate	1 (1.6)	62 (98.4)		38 (60.3)	25 (39.7)		39 (61.9)	24 (38.1)		29 (46.0)	34 (54.0)	
Marital status	Single, div/wid ^1^	2 (1.0)	197 (99.0)	0.993	123 (61.8)	76 (38.2)	0.823	135 (67.8)	64 (32.2)	0.149	126 (63.3)	73 (36.7)	**<0.001**
	Married	3 (1.0)	293 (99.0)		180 (60.8)	116 (39.2)		182 (61.5)	114 (38.5)		121 (40.9)	175 (59.1)	
Self-reported financial status	Poor or fair	3 (1.3)	225 (98.7)	0.530	142 (62.3)	86 (37.7)	0.652	153 (67.1)	75 (32.9)	0.189	117 (51.3)	111 (48.7)	0.560
	Good or excellent	2 (0.7)	265 (99.3)		161 (60.3)	106 (39.7)		164 (61.4)	103 (38.6)		130 (48.7)	137 (51.3)	
Residence	Amman	3 (1.1)	281 (98.9)	0.905	161 (56.7)	123 (43.3)	**0.017**	170 (59.9)	114 (40.1)	**0.025**	138 (48.6)	146 (51.4)	0.500
	Outside the Capital	2 (0.9)	209 (99.1)		142 (67.3)	69 (32.7)		147 (69.7)	64 (30.3)		109 (51.7)	102 (48.3)	
Occupational category	Nurse	4 (1.3)	298 (98.7)	0.382	193 (63.9)	109 (36.1)	0.124	188 (62.3)	114 (37.7)	0.300	128 (42.4)	174 (57.6)	**<0.001**
	Physician	1 (0.5)	192 (99.5)		110 (57.0)	83 (43.0)		129 (66.8)	64 (33.2)		119 (61.7)	74 (38.3)	
Seniority level	1–9 years	4 (1.4)	281 (98.6)	0.308	180 (63.2)	105 (36.8)	0.301	198 (69.5)	87 (30.5)	**0.003**	173 (60.7)	112 (39.3)	**<0.001**
	10 years or more	1 (0.5)	209 (99.5)		123 (58.6)	87 (41.4)		119 (56.7)	91 (43.3)		74 (35.2)	136 (64.8)	
Nationality	Jordanian	5 (1.0)	473 (99.0)	0.672	296 (61.9)	182 (38.1)	0.084	309 (64.6)	169 (35.4)	0.138	239 (50.0)	239 (50.0)	0.812
	Non-Jordanian	0	17 (100)		7 (41.2)	10 (58.8)		8 (47.1)	9 (52.9)		8 (47.1)	9 (52.9)	
Self-reported history of chronic disease	Yes	0	51 (100)	0.446	26 (51.0)	25 (49.0)	0.113	28 (54.9)	23 (45.1)	0.151	19 (37.3)	32 (62.7)	0.057
	No	5 (1.1)	439 (98.9)		277 (62.4)	167 (37.6)		289 (65.1)	155 (34.9)		228 (51.4)	216 (48.6)	

^1^ div/wid: divorced, widow/widower; ^2^ COVID-19: Coronavirus disease 2019; ^3^ *p* values were calculated using chi-squared test. Statistically significant *p* values are highlighted in bold style.

**Table 5:** Intention to get Mpox vaccination in relation with different study variables.

Variable	Category	If a Safe and Effective Mpox Vaccine Is Available Free of Charge, Would You Be Willing to Receive It?			*p* Value ^5^
		Yes N ^4^ (%)	Maybe N (%)	No N (%)	
Age	≤32 years	82 (33.5)	91 (37.1)	72 (29.4)	**0.001**
	>32 years	61 (24.4)	74 (29.6)	115 (46.0)	
Sex	Male	64 (31.4)	65 (31.9)	75 (36.8)	0.588
	Female	79 (27.1)	100 (34.4)	112 (38.5)	
Highest educational level	Undergraduate	121 (28.0)	147 (34.0)	164 (38.0)	0.489
	Postgraduate	22 (34.9)	18 (28.6)	23 (36.5)	
Marital status	Single, divorced, widow/widower	71 (35.7)	80 (40.2)	48 (24.1)	**<0.001**
	Married	72 (24.3)	85 (28.7)	139 (47.0)	
Self-reported financial status	Poor or fair	61 (26.8)	81 (35.5)	86 (37.7)	0.528
	Good or excellent	82 (30.7)	84 (31.5)	101 (37.8)	
Residence	Amman	98 (34.5)	95 (33.5)	91 (32.0)	**0.001**
	Outside the Capital	45 (21.3)	70 (33.2)	96 (45.5)	
Occupational category	Nurse	70 (23.2)	91 (30.1)	141 (46.7)	**<0.001**
	Physician	73 (37.8)	74 (38.3)	46 (23.8)	
Seniority level	1–9 years	89 (31.2)	111 (38.9)	85 (29.8)	**<0.001**
	10 years or more	54 (25.7)	54 (25.7)	102 (48.6)	
Nationality	Jordanian	135 (28.2)	159 (33.3)	184 (38.5)	0.141
	Non-Jordanian	8 (47.1)	6 (35.3)	3 (17.6)	
Self-reported history of chronic disease	Yes	15 (29.4)	13 (25.5)	23 (45.1)	0.394
	No	128 (28.8)	152 (34.2)	164 (36.9)	
Mpox K score ^1^	≤12	50 (29.1)	52 (30.2)	70 (40.7)	0.142
	>12	80 (31.0)	96 (37.2)	82 (31.8)	
VB score ^2^	≤2	64 (21.6)	102 (34.5)	130 (43.9)	**<0.001**
	>2	79 (39.7)	63 (31.7)	57 (28.6)	
5C scale ^3^	≤26	21 (9.5)	58 (26.4)	141 (64.1)	**<0.001**
	>26	122 (44.4)	107 (38.9)	46 (16.7)	

^1^ Mpox K score: Monkeypox knowledge score calculated based on 10 knowledge items. Please notice that this score was only calculated for the participants who had heard of Mpox prior to the study; therefore, the number of respondents with a valid Mpox K score will not add up to the total number of study respondents; ^2^ VP score: Previous vaccine behavior score calculated based on self-reported uptake of influenza vaccine, primary and booster COVID-19 vaccination; ^3^ 5C scale: Calculated based on nine items adopted to assess the psychological determinants of vaccination; ^4^ N: Number; ^5^ *p* values were calculated using chi-squared test. Statistically significant *p* values are highlighted in bold style.

**Table 6:** The association of different study variables with each of the 5C scale items.

Variable	Category	Confidence 1 Mean ± SD	Confidence 2 Mean ± SD	Collective Responsibility	Complacency 1 Mean ± SD	Complacency 2 Mean ± SD	Constraints 1 Mean ± SD	Constraints 2 Mean ± SD	Calculation 1 Mean ± SD	Calculation 2 Mean ± SD	5C Mean ± SD
Age	≤32 years	3.37 ± 1.00	3.41 ± 1.00	3.74 ± 0.90	3.40 ± 0.90	3.43 ± 0.87	3.06 ± 1.05	3.09 ± 1.09	1.87 ± 0.83	1.81 ± 0.80	27.18 ± 4.90
	>32 years	3.01 ± 1.10	3.05 ± 1.07	3.50 ± 1.13	3.13 ± 0.93	3.23 ± 0.87	2.88 ± 1.14	2.97 ± 1.12	2.05 ± 0.94	1.87 ± 0.92	25.69 ± 4.76
	*p* value ^1^	**<0.001**	**<0.001**	**0.020**	**0.001**	**0.011**	0.135	0.209	**0.035**	0.783	**<0.001**
Sex	Male	3.22 ± 1.09	3.27 ± 1.07	3.62 ± 1.07	3.22 ± 0.97	3.24 ± 0.90	2.87 ± 1.13	3.01 ± 1.12	1.96 ± 0.87	1.84 ± 0.85	26.25 ± 5.34
	Female	3.17 ± 1.05	3.20 ± 1.04	3.61 ± 1.00	3.29 ± 0.89	3.40 ± 0.85	3.04 ± 1.07	3.04 ± 1.10	1.96 ± 0.90	1.84 ± 0.87	26.55 ± 4.54
	*p* value	0.578	0.444	0.712	0.437	**0.039**	0.094	0.731	0.887	0.870	0.669
Education	Undergraduate	3.18 ± 1.08	3.22 ± 1.06	3.62 ± 1.05	3.27 ± 0.93	3.33 ± 0.88	2.99 ± 1.11	3.03 ± 1.12	1.98 ± 0.90	1.84 ± 0.87	26.46 ± 4.94
	Postgraduate	3.24 ± 0.95	3.29 ± 0.99	3.59 ± 0.89	3.22 ± 0.89	3.37 ± 0.83	2.84 ± 1.03	3.00 ± 1.05	1.84 ± 0.79	1.86 ± 0.80	26.24 ± 4.43
	*p* value	0.702	0.691	0.562	0.677	0.796	0.208	0.660	0.361	0.673	0.571
Marital status	Single/div/wid ^2^	3.46 ± 1.02	3.47 ± 1.01	3.89 ± 0.90	3.46 ± 0.91	3.48 ± 0.86	3.06 ± 1.09	3.13 ± 1.13	1.80 ± 0.80	1.74 ± 0.75	27.49 ± 5.03
	Married	3.01 ± 1.06	3.07 ± 1.05	3.43 ± 1.06	3.13 ± 0.91	3.23 ± 0.87	2.91 ± 1.10	2.96 ± 1.08	2.06 ± 0.93	1.91 ± 0.92	25.72 ± 4.65
	*p* value	**<0.001**	**<0.001**	**<0.001**	**<0.001**	**0.002**	0.113	**0.044**	**0.002**	0.086	**<0.001**
Financial status	Poor/fair	3.14 ± 1.05	3.19 ± 1.04	3.56 ± 1.02	3.17 ± 0.93	3.26 ± 0.87	2.91 ± 1.10	2.98 ± 1.10	2.00 ± 0.90	1.92 ± 0.88	26.11 ± 4.75
	Good/excellent	3.24 ± 1.08	3.27 ± 1.07	3.66 ± 1.03	3.34 ± 0.91	3.39 ± 0.87	3.02 ± 1.09	3.07 ± 1.11	1.93 ± 0.88	1.78 ± 0.84	26.70 ± 4.98
	*p* value	0.292	0.386	0.258	**0.011**	**0.022**	0.194	0.209	0.398	0.076	0.193
Residence	Amman	3.32 ± 1.03	3.37 ± 1.02	3.72 ± 0.95	3.41 ± 0.82	3.46 ± 0.78	3.10 ± 1.07	3.20 ± 1.06	1.91 ± 0.87	1.80 ± 0.82	27.29 ± 4.61
	Out. Amman ^3^	3.01 ± 1.08	3.04 ± 1.07	3.47 ± 1.11	3.06 ± 1.01	3.16 ± 0.96	2.79 ± 1.11	2.80 ± 1.13	2.02 ± 0.91	1.91 ± 0.92	25.27 ± 5.00
	*p* value	**0.002**	**0.001**	**0.017**	**<0.001**	**0.001**	**0.004**	**<0.001**	0.170	0.269	**<0.001**
Occupation	Nurse	3.00 ± 1.07	3.05 ± 1.06	3.45 ± 1.09	3.09 ± 0.94	3.19 ± 0.89	2.90 ± 1.13	2.91 ± 1.10	2.09 ± 0.94	1.92 ± 0.93	25.58 ± 4.70
	Physician	3.49 ± 0.98	3.52 ± 0.98	3.88 ± 0.86	3.53 ± 0.82	3.56 ± 0.79	3.07 ± 1.05	3.22 ± 1.09	1.76 ± 0.77	1.73 ± 0.72	27.77 ± 4.87
	*p* value	**<0.001**	**<0.001**	**<0.001**	**<0.001**	**<0.001**	0.153	**0.001**	**<0.001**	0.082	**<0.001**
Seniority	1–9 years	3.32 ± 1.01	3.39 ± 1.01	3.74 ± 0.92	3.39 ± 0.9	3.42 ± 0.84	2.99 ± 1.07	3.06 ± 1.10	1.86 ± 0.82	1.79 ± 0.80	26.94 ± 4.84
	≥10 years	3.02 ± 1.11	3.02 ± 1.08	3.45 ± 1.14	3.10 ± 0.93	3.20 ± 0.9	2.94 ± 1.14	3.00 ± 1.12	2.10 ± 0.96	1.92 ± 0.94	25.74 ± 4.85
	*p* value	**0.002**	**<0.001**	**0.004**	**0.001**	**0.011**	0.930	0.606	**0.007**	0.224	**0.006**
Nationality	Jordanian	3.17 ± 1.06	3.21 ± 1.05	3.60 ± 1.03	3.26 ± 0.91	3.32 ± 0.88	2.96 ± 1.10	3.03 ± 1.11	1.96 ± 0.89	1.84 ± 0.87	26.37 ± 4.86
	Non-Jordanian	3.65 ± 1.06	3.82 ± 1.13	3.94 ± 1.03	3.24 ± 1.15	3.53 ± 0.72	3.06 ± 1.03	3.12 ± 1.05	1.88 ± 0.70	1.88 ± 0.70	28.12 ± 5.18
	*p* value	0.052	**0.014**	0.127	0.847	0.448	0.710	0.722	0.911	0.597	0.069
Chronic disease	Yes	2.90 ± 1.08	3.00 ± 1.00	3.43 ± 1.08	3.10 ± 0.96	3.22 ± 0.88	2.98 ± 1.07	3.06 ± 1.01	2.27 ± 0.92	2.12 ± 0.95	26.08 ± 4.48
	No	3.22 ± 1.06	3.26 ± 1.06	3.64 ± 1.02	3.28 ± 0.92	3.34 ± 0.87	2.97 ± 1.10	3.03 ± 1.12	1.92 ± 0.88	1.81 ± 0.84	26.47 ± 4.93
	*p* value	0.051	0.124	0.176	0.196	0.305	0.936	0.994	**0.007**	**0.023**	0.419

^1^*p* Values were calculated using the Mann–Whitney *U* test; ^2^ div/wid: divorced, widow/widower; ^3^ Out. Amman: Outside the Capital. Statistically significant *p* values are highlighted in bold style.

**Table 7:** Factors associated with higher intention to receive monkeypox (Mpox) vaccination.

5C Item	Response	Willingness to Get Mpox Vaccination			*p* Value ^1^
		Acceptance Group	Hesitancy Group	Resistance Group	
I will receive Mpox vaccination if it is effective	Agreement	128 (89.5)	61 (37.0)	12 (6.4)	**<0.001**
	Neutral	12 (8.4)	92 (55.8)	47 (25.1)	
	Disagreement	3 (2.1)	12 (7.3)	128 (68.4)	
I will receive Mpox vaccination if the Ministry of Health recommends the vaccine	Agreement	120 (83.9)	68 (41.2)	20 (10.7)	**<0.001**
	Neutral	18 (12.6)	83 (50.3)	51 (27.3)	
	Disagreement	5 (3.5)	14 (8.5)	116 (62.0)	
It is important that I get Mpox vaccination to protect community members with weaker immunity	Agreement	121 (84.6)	98 (59.4)	54 (28.9)	**<0.001**
	Neutral	19 (13.3)	61 (37.0)	72 (38.5)	
	Disagreement	3 (2.1)	6 (3.6)	61 (32.6)	
I will not receive Mpox vaccination because I have a strong immune system which will protect me from the disease	Disagreement	99 (69.2)	82 (49.7)	49 (26.2)	**<0.001**
	Neutral	29 (20.3)	68 (41.2)	76 (40.6)	
	Agreement	15 (10.5)	15 (9.1)	62 (33.2)	
I will not receive Mpox vaccination because the disease is not dangerous	Disagreement	104 (72.7)	80 (48.5)	55 (29.4)	**<0.001**
	Neutral	25 (17.5)	76 (46.1)	81 (43.3)	
	Agreement	14 (9.8)	9 (5.5)	51 (27.3)	
I would not receive Mpox vaccination if I had to pay for the vaccine	Disagreement	70 (49.0)	62 (37.6)	56 (29.9)	**<0.001**
	Neutral	35 (24.5)	66 (40)	54 (28.9)	
	Agreement	38 (26.6)	37 (22.4)	77 (41.2)	
I will not receive Mpox vaccination if I have to register on online platforms or wait for a long time	Disagreement	83 (58.0)	70 (42.4)	59 (31.6)	**<0.001**
	Neutral	24 (16.8)	60 (36.4)	49 (26.2)	
	Agreement	36 (25.2)	35 (21.2)	79 (42.2)	
Before receiving the vaccine, it is important that I weigh the benefits and potential harm of the vaccine	Disagreement	5 (3.5)	4 (2.4)	21 (11.2)	**<0.001**
	Neutral	18 (12.6)	33 (20.0)	41 (21.9)	
	Agreement	120 (83.9)	128 (77.6)	125 (66.8)	
It is important that I fully understand all about Mpox vaccination before I decide to receive it	Disagreement	2 (1.4)	4 (2.4)	17 (9.1)	**0.001**
	Neutral	16 (11.2)	30 (18.2)	32 (17.1)	
	Agreement	125 (87.4)	131 (79.4)	138 (73.8)	

^1^*p* Values were calculated using chi-squared test. Statistically significant *p* values are highlighted in bold style.

**Table 8:** Factors associated with higher intention to receive Mpox vaccination.

Mpox Vaccine Acceptance vs. Mpox Vaccine Hesitancy	OR (95% CI) ^3^	*p* Value
Age: >32 years vs. ≤32 years	0.845 (0.382–1.870)	0.678
Marital status: Married vs. single, divorced, widow/widower	0.987 (0.555–1.755)	0.964
Residence: Outside the Capital vs. Amman	0.731 (0.442–1.211)	0.224
Occupational category: Physician vs. nurse	1.326 (0.708–2.484)	0.378
Seniority level: 10 years or more vs. 1–9 years	1.406 (0.643–3.075)	0.742
VB score ^1^: >2 vs. ≤2	1.980 (1.208–3.244)	**0.007**
5C scale ^2^: >26 vs. ≤26	2.972 (1.675–5.272)	**<0.001**
**Mpox Vaccine Acceptance vs. Mpox Vaccine Rejection**	**OR (95% CI)**	***p* Value**
Age: >32 years vs. ≤32 years	1.638 (0.689–3.891)	0.264
Marital status: Married vs. single, divorced, widow/widower	0.429 (0.221–0.834)	**0.013**
Residence: Outside the Capital vs. Amman	0.610 (0.349–1.065)	0.082
Occupational category: Physician vs. nurse	1.688 (0.827–3.442)	0.150
Seniority level: 10 years or more vs. 1–9 years	0.514 (0.221–1.193)	0.121
VB score: >2 vs. ≤2	3.860 (2.180–6.835)	**<0.001**
5C scale: >26 vs. ≤26	16.129 (8.868–29.336)	**<0.001**

^1^ VP score: Previous vaccine behavior score was calculated based on self-reported uptake of influenza vaccine, primary and booster COVID-19 vaccination; ^2^ 5C scale: Calculated based on nine items adopted to assess the psychological determinants of vaccination; ^3^ OR: Odds ratio, CI: Confidence interval. Statistically significant *p* values are highlighted in bold style.

**Table 9:** The overall attitude towards compulsory vaccination. stratified by occupational category.

Item	Response	Nurse N ^4^ (%)	Physician N (%)	*p* Value ^5^
COVID-19 ^1^ vaccination should be compulsory for all members of society	Strongly agree	43 (14.2)	35 (18.1)	0.305
	Agree	68 (22.5)	43 (22.3)	
	Neutral/no opinion	65 (21.5)	51 (26.4)	
	Disagree	106 (35.1)	52 (26.9)	
	Strongly disagree	20 (6.6)	12 (6.2)	
COVID-19 vaccination should be compulsory for all HCWs ^2^	Strongly agree	62 (20.5)	56 (29.0)	**0.017**
	Agree	81 (26.8)	62 (32.1)	
	Neutral/no opinion	60 (19.9)	36 (18.7)	
	Disagree	82 (27.2)	30 (15.5)	
	Strongly disagree	17 (5.6)	9 (4.7)	
Influenza vaccination should be compulsory for all members of society	Strongly agree	35 (11.6)	27 (14.0)	0.253
	Agree	70 (23.2)	29 (15.0)	
	Neutral/no opinion	78 (25.8)	57 (29.5)	
	Disagree	100 (33.1)	69 (35.8)	
	Strongly disagree	19 (6.3)	11 (5.7)	
Influenza vaccination should be compulsory for all HCWs	Strongly agree	48 (15.9)	43 (22.3)	0.347
	Agree	74 (24.5)	47 (24.4)	
	Neutral/no opinion	71 (23.5)	47 (24.4)	
	Disagree	92 (30.5)	48 (24.9)	
	Strongly disagree	17 (5.6)	8 (4.1)	
Mpox ^3^ vaccination should be compulsory for all members of society	Strongly agree	20 (6.6)	19 (9.8)	**0.001**
	Agree	45 (14.9)	12 (6.2)	
	Neutral/no opinion	83 (27.5)	78 (40.4)	
	Disagree	133 (44.0)	65 (33.7)	
	Strongly disagree	21 (7.0)	19 (9.8)	
Mpox vaccination should be compulsory for all HCWs	Strongly agree	27 (8.9)	23 (11.9)	**0.047**
	Agree	52 (17.2)	28 (14.5)	
	Neutral/no opinion	84 (27.8)	73 (37.8)	
	Disagree	120 (39.7)	55 (28.5)	
	Strongly disagree	19 (6.3)	14 (7.3)	

^1^ COVID-19: Coronavirus disease 2019; ^2^ HCWs: Healthcare workers; ^3^ Mpox: Monkeypox; ^4^ N: Number; ^5^ *p* value: Calculated using chi-squared test. Statistically significant *p* values are highlighted in bold style.

**Table 10:** Variables associated with attitude towards compulsory vaccination.

Variable	Category	Attitude to Compulsory Vaccination		*p* Value ^5^
		≤18 N ^4^ (%)	>18 N (%)	
Age	≤32 years	130 (53.1)	115 (46.9)	0.198
	>32 years	147 (58.8)	103 (41.2)	
Sex	Male	114 (55.9)	90 (44.1)	0.977
	Female	163 (56.0)	128 (44.0)	
Highest educational level	Undergraduate	236 (54.6)	196 (45.4)	0.119
	Postgraduate	41 (65.1)	22 (34.9)	
Marital status	Single, divorced, widow/widower	108 (54.3)	91 (45.7)	0.535
	Married	169 (57.1)	127 (42.9)	
Self-reported financial status	Poor or fair	128 (56.1)	100 (43.9)	0.940
	Good or excellent	149 (55.8)	118 (44.2)	
Residence	Amman	149 (52.5)	135 (47.5)	0.069
	Outside the Capital	128 (60.7)	83 (39.3)	
Occupational category	Nurse	176 (58.3)	126 (41.7)	0.194
	Physician	101 (52.3)	92 (47.7)	
Seniority level	1–9 years	150 (52.6)	135 (47.4)	0.082
	10 years or more	127 (60.5)	83 (39.5)	
Nationality	Jordanian	268 (56.1)	210 (43.9)	0.799
	Non-Jordanian	9 (52.9)	8 (47.1)	
Self-reported history of chronic disease	Yes	35 (68.6)	16 (31.4)	0.054
	No	242 (54.5)	202 (45.5)	
Mpox K score ^1^	≤12	91 (52.9)	81 (47.1)	0.406
	>12	147 (57.0)	111 (43.0)	
VB score ^2^	≤2	184 (62.2)	112 (37.8)	**0.001**
	>2	93 (46.7)	106 (53.3)	
5C score ^3^	≤26	152 (69.1)	68 (30.9)	**<0.001**
	>26	125 (45.5)	150 (54.5)	

^1^ Mpox K score: Monkeypox knowledge score calculated based on 10 knowledge items. Please notice that this score was only calculated for the participants who had heard of Mpox prior to the study; therefore, the number of respondents with a valid Mpox K score will not add up to the total number of study respondents; ^2^ VP score: Previous vaccine behavior score calculated based on self-reported uptake of influenza vaccine, primary and booster COVID-19 vaccination; ^3^ 5C scale: Calculated based on nine items adopted to assess the psychological determinants of vaccination; ^4^ N: Number; ^5^ *p* values were calculated using chi-squared test. Statistically significant *p* values are highlighted in bold style.

**Table 11:** Factors associated with favorable attitudes towards compulsory vaccination.

Agreement vs. Neutral/Disagreement towards Compulsory Vaccination	OR (95% CI) ^3^	*p* Value
Residence: Outside the Capital vs. Amman	0.821 (0.563–1.198)	0.307
Seniority level: 10 years or more vs. 1–9 years	0.681 (0.460–1.009)	0.056
VB score ^1^: >2 vs. ≤2	1.930 (1.306–2.853)	**0.001**
5C scale ^2^: >26 vs. ≤26	2.430 (1.662–3.554)	**<0.001**

^1^ VP score: Previous vaccine behavior score calculated based on self-reported uptake of influenza vaccine, primary and booster COVID-19 vaccination; ^2^ 5C scale: Calculated based on nine items adopted to assess the psychological determinants of vaccination; ^3^ OR: Odds ratio, CI: Confidence interval. Statistically significant *p* values are highlighted in bold style.

## Data Availability

Data presented in the current study are available upon request from the corresponding authors (M.S.).
